# Mortality after Fluid Bolus in Children with Shock Due to Sepsis or Severe Infection: A Systematic Review and Meta-Analysis

**DOI:** 10.1371/journal.pone.0043953

**Published:** 2012-08-30

**Authors:** Nathan Ford, Sally Hargreaves, Leslie Shanks

**Affiliations:** 1 Médecins Sans Frontières, Geneva, Switzerland; 2 Centre for Infectious Disease Epidemiology and Research, University of Cape Town, Cape Town, South Africa; 3 The International Health Unit, Department of Infectious Diseases and Immunity, Hammersmith Hospital, Imperial College London, London, United Kingdom; 4 Médecins Sans Frontières, Amsterdam, The Netherlands; Menzies School of Health Research, Australia

## Abstract

**Introduction:**

Sepsis is one of the leading causes of childhood mortality, yet controversy surrounds the current treatment approach. We conducted a systematic review to assess the evidence base for fluid resuscitation in the treatment of children with shock due to sepsis or severe infection.

**Methods:**

We searched 3 databases for randomized trials, quasi-randomized trials, and controlled before-after studies assessing children with septic shock in which at least one group was treated with bolus fluids. The primary outcome was mortality at 48 hours. Assessment of methodological quality followed the GRADE criteria. Relative risks (RRs) and 95% confidence intervals (CI) were calculated and data pooled using fixed-effects method.

**Results:**

13 studies met our inclusion criteria. No bolus has significantly better mortality outcomes at 48 hours for children with general septic shock (RR 0.69; 95%CI 0.54–0.89), and children with malaria (RR 0.64; 95%CI 0.45–0.91) when compared to giving any bolus. This result is largely driven by a single, high quality trial (the FEAST trial). There is no evidence investigating bolus vs no bolus in children with Dengue fever or severe malnutrition. Colloid and crystalloid boluses were found to have similar effects on mortality across all sub-groups (general septic shock, malaria, Dengue fever, and severe malnutrition).

**Conclusions:**

The majority of all randomized evidence to date comes from the FEAST trial, which found that fluid boluses were harmful compared to no bolus. Simple algorithms are needed to support health-care providers in the triage of patients to determine who could potentially be harmed by the provision of bolus fluids, and who will benefit.

## Introduction

Sepsis is one of the leading causes of childhood mortality, responsible for over half a million deaths world-wide [1]. Early rapid fluid therapy is part of the standard package of care for children with septic shock [2], [3]. Despite decades of concern and numerous practice guidelines, the use of fluid resuscitation in the management of paediatric septic shock has, until recently, been based on limited evidence. Recommendations to date have been derived largely from experience of treating septic shock in adults [4], and until recently were supported by data from non-comparative cohorts of ionotrope-dependant children in a tertiary care setting [5], [6].

A recent systematic review that assessed differences in choice of resuscitation fluids (colloid vs crystalloids) concluded that there was insufficient evidence to make a definitive choice of fluids given the weak evidence base [7]. However, this review did not look at the question of whether or not fluid resuscitation improves outcomes. Several trials have since been published, most notably a large randomized-controlled trial, the FEAST trial, which found that fluid bolus in fact increased mortality compared to no fluid bolus [8]. Despite the large effect size of this trial, the results have led to considerable controversy regarding the applicability of the trial results to different contexts and populations [9]–[14], and to date no revisions have been made to international and national guidelines to reflect new trial findings.

We conducted a systematic review to assess the current evidence base for fluid resuscitation in the treatment of children with shock due to sepsis or severe infection.

## Methods

### Search Strategy

Our systematic review was conducted in accordance with the PRISMA guidelines for reporting systematic reviews and meta-analyses [15].

Three databases – MEDLINE via PubMed, EMBASE, and the Cochrane Central Register of Controlled Trials (CENTRAL) – were searched independently and in duplicate by 2 reviewers (SH, NF) from inception to February 29, 2012 with no geographical or language restrictions using a compound search strategy detailed in the pre-defined protocol (File S1). We additionally searched bibliographies of relevant reviews and contacted experts in the field in an attempt to identify relevant studies. Data extraction was done independently and in duplicate by two reviewers (NF, SH). The review sought randomized trials, quasi-randomized trials, and controlled before-after studies assessing children with septic shock and/or shock and severe infection (as defined by the studies) in which at least one group was treated with bolus fluids. Studies that only addressed non-infectious causes of shock, neonatal shock, or patient populations with severe dehydration, were excluded consistent with previous systematic reviews [7]. Studies in which >30% of participants were considered to have septic shock were included, but outcomes were not pooled. The primary outcome was mortality at 48 hours. Secondary outcomes included mortality at 4 weeks and adverse clinical events. Results were pooled according to cause of septic shock.

### Assessment of Methodological Quality

Individual studies were rated according to three main indicators of methodological quality of randomized trials: allocation concealment, loss to follow up <20%, and reporting of adverse events. For each category of septic shock, assessment of methodological quality followed GRADE which rates evidence according to four criteria: limitations, inconsistency, indirectness, and imprecision. Publication bias was considered as a potential limitation of the systematic review overall.

### Data Analysis

Relative risks (RRs) and 95% confidence intervals (CI) were calculated and data pooled using fixed-effects method, in which the weight assigned the estimated treatment effect from a given trial is proportional to the amount of information provided by that trial. The robustness of this analysis was explored in sensitivity analysis using the random-effects method [16]. Data were pooled according to pre-defined subgroups depending on cause of sepsis given differences in prognosis, and heterogeneity estimated by the *I* ^2^ statistic. Point estimates and 95% CIs were calculated for the frequencies of adverse events. All analyses were conducted using Stata, version 12 (StataCorp LP, College Station, Texas, USA) and GRADE Pro (www.gradeworkinggroup.org).

## Results

### Study Inclusions

**Figure 1:**
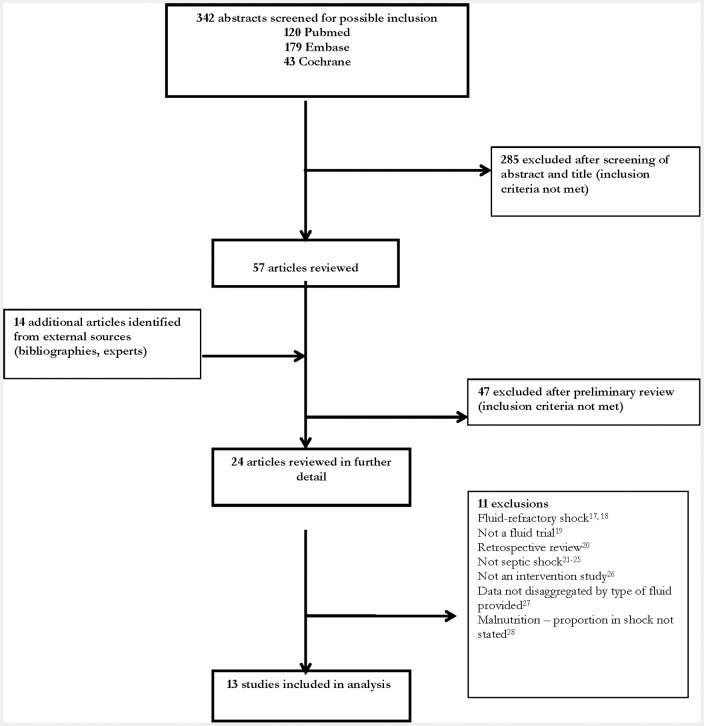
Study inclusions and exclusions.

The search strategy yielded 342 articles that were screened by title and abstract. An additional 14 articles were identified through bibliographic searches and contact with experts. In total, 13 studies met the inclusion criteria and were taken through for full review [8], [17]–[28]. Study inclusions as well as final exclusions are detailed in Figure 1. Studies were done in populations with malaria (4 studies), dengue fever (4 studies), and mixed causes of septic shock (4 studies). In addition, one study was done in children with severe malnutrition, in which over a third (34%) were determined to have hypovolemic shock secondary to sepsis. Baseline characteristics of included studies are summarised in Table 1.

**Table 1:** Study characteristics.

Study	Setting	Population	Sample size	Inclusions	Exclusions	Definition of shock	Intervention
Maitland et al,2011 [8] i	KenyaTanzaniaUganda	Children aged >2months with severefebrile illness	3141	Severe febrile illness with either impaired consciousness, and/or respiratory distress	Severe malnutritionGastroenteritisNon-infectious shockContra-indications:Severe hypotension: systolic blood pressure <50mmHg if <12 months; <60mmHg if 1–5 years; <70mmHg if >5 years	Impaired perfusion (defined as one or more of capillary refilling time (CRT) ≥3 secs, severe tachycardia, temperature gradient or weak pulse	20–40ml/kg over the first hour: 5% albumin vs 0.9% saline vs no bolus(control)
Chopra et al,2011 [17]	India	Children 2–12 yearswith Septic shock	60	Children with septic shock	Critical status (ARDS, immunodeficiency, severe PEM)	Sepsis and hypotension (not defined) or sepsis plus 3 out 4 signs of hypoperfusion, decreased pulse volume, CRT≥3 secs, tachycardia,urine output<1 ml/kg/h.	0.9% saline vs 3% saline
Santhanam et al,2008 [18]	India	Children aged1 month - 12 yearswith Septic shock	147	Children with septic shock	Various	Sepsis ConsensusConference definition	Different volumes & durations of Ringers lactate + dopamine if therapeutic goals not attained
Upadhyay et al,2005 [19] ii	India	Children aged1 month - 12 yearswith Septic shock	60	Children with septic shock	Critical status (multi-organ failure, underlying immunodeficiency)	Sepsis and hypotension (not defined) OR sepsis with 3 out of four signs of hypoperfusion decreased pulse volume, CRT ≥3secs, tachycardia, urine output <1ml/kg/hr	Degraded gelatin vs normal saline
Akech et al,2010b [21] iii	Kenya	Children >6 monthswith severe malaria	79	Severe malaria *P. falciparum* parasitaemia, plus impaired consciousness and/or deep breathing plus metabolic acidosis (base excess greater than - 8)	Haemoglobin <5g/dl, pulmonary oedema, congenital heart disease, severemalnutrition, or unable to defer consent: decompensateshock	CRT ≥3 secs, hypoxia (O_2_ sat ≤95%) tachycardia (180bpm if <12months, 160 bpm if aged 1–5 yrs, 140 bpm if >5 yrs); or hypotension (SBP<70 mm Hg if <12 months or <80 mm Hg if >1 yr	20 ml/kg over the first hour, repeat if still in shock: 6% Dextran 70 vs 6% hydroxyethyl starch
Akech et al,2006 [20] iv	Kenya	Severe Malaria(Children >6 months)	88	Severe malaria *P. falciparum* parasitaemia, plus impaired consciousness and/or deep breathing plus metabolic acidosis (base excess greater than - 8)	Pulmonary oedema, oedematousmalnutrition, papilloedema	CRT ≥3 secs, hypoxia (O_2_ sat ≤95%) tachycardia (180bpm if <12months, 160 bpm if aged 1–5 yrs, 140 bpm if >5 yrs); or hypotension (SBP<70 mm Hg if <12 months or <80 mm Hg if >1 yr	20 ml/kg over the first hour, repeat if in shock: 4.5% albumin vs Gelofusine
Maitland et al,2005 [23] a	Kenya	Severe Malaria(Children >6 months)	150	Severe malaria *P. falciparum* parasitaemia, plus impaired consciousness and/or deep breathing plus metabolic acidosis (base excess greater than - 8)	Pulmonary oedema, oedematous malnutrition, papilledema	Shock not an inclusion criteria, only assumed as main cause of metabolic acidosis	4.5% albumin vs 0.9% saline vs control (4% dextrose/0.18% saline)
Maitland et al,2005 [22] b	Kenya	Severe MalarialAnaemia(children >2 months)	61	Severe malaria anaemia : *P. falciparum,* deep breathing and haemoglobin <5g/dl plus metabolic acidosis (base excess greater than - 8)	Pulmonary oedema, oedematous kwashiorkor, papillodema, severe anaemia from another cause	Criteria for rescue:Hypotension (SBP<70 or <80 mmHg in children>1 year); sustained oliguria (urine output <1 ml/kg/h);	Pre-transfusion management of 20 ml/kg over the first hour: 0.9% saline vs 4.5% albumin vs no bolus (maintenance-only)
Wills et al2005 [28]	Vietnam	Dengue	512	Clinical dengue shock syndrome	None	WHO guidelines	Moderate: Ringers lactate, dextran, or starch; Severe: dextran/starch
Cifra et al,2003 [24]	Philippines	Dengue	27	Dengue Shock syndrome	Other severe infection, protein-deficient abnormalities, bleeding diathesis, patients who have been given multiple plasma substitutes		Hydroxyethyl starch vs Ringers lactate
Ngo et al,2001 [27]	Vietnam	Dengue, aged5–15 years and	230	Children withDengue shocksyndrome,	Severe haemmorhage requiring transfusion; not received IV fluid therapy during current illness	(pulse pressure ≤20mmHg; low cardiac output	Dextran 70 vs 3% Gelatin vs Ringers lactate saline
Dung et al,1999 [26]	Vietnam	Dengue aged5–15 years	50	Dengue shocksyndrome;	Not received fluids in this illness	DHF with either low pulse pressure ((pulse pressure ≤20mmHg), or unrecordable blood pressure + clinical signs of circulatory insufficiency.	Dextran 70 vs Gelafundin vs Ringers lactate vs 0.9% saline
Akech et al,2010a [25] v	Kenya	SevereMalnutrition	61	Severe malnutrition with septic shock orSevere dehydrating shock	Severe anaemia; pulmonary oedema;raised intracranial pressure or CHD	Amended WHO malnutrition shockCriteria (CRT ≥3 secs, weak pulse volume, temperature gradient, deepbreathing, creatinine >80 µmol/L, depressed level of consciousness	15 ml/kg over the first hour: half strength Ringers/5% Dextrose (15 ml/kg over two hours) vs Ringers lactate vs 4.5% albumin (10ml/kg over 30 mins, repeat up to 3 times)

i 57% (1793) positive for malaria;

ii 8% (53) had evidence of infection;

iii only 1 child had evidence of bacterial sepsis;

iv >80% met definition for shock;

v 34% (21) had hypovolemic shock secondary to sepsis.

### Assessment of Methodological Quality

The assessment of methodological quality of individual studies is summarised in File S2. Overall, the methodological quality of trials was high: most studies used allocation concealment (10/14) and reported adverse events (12/14), and all had low rates of loss-to-follow up. The GRADE evidence assessment for each category of shock trial is summarised in File S3. For septic shock the quality of the evidence was rated as high; this rating was driven largely by the contribution of the FEAST trial. For malaria the quality of the evidence was rated high if the FEAST trial data was considered, otherwise it was rated as moderate, mainly due to imprecision. For Dengue the quality of the evidence was rated as moderate; this was due to the rating of serious imprecision driven by the low event rate in trials. For malnutrition the quality of evidence was graded as low because there was only a single, small trial.

### Primary Outcomes

Primary outcomes of mortality at 48 hours are summarised in Figure 1.

**Figure 2:**
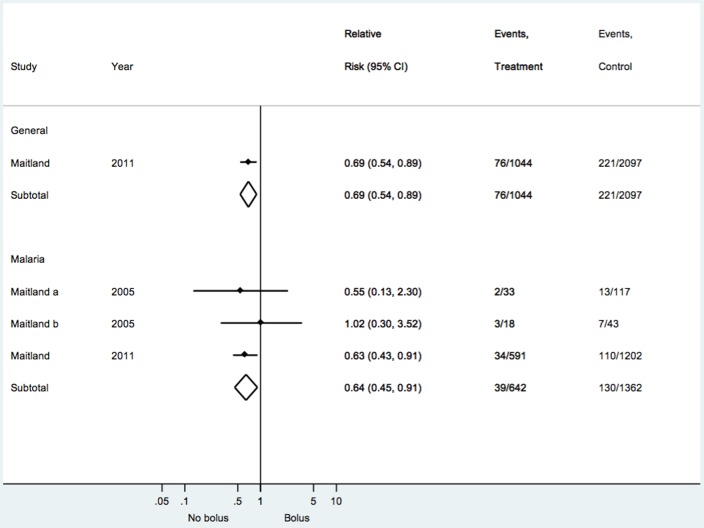
Forest plot for the outcome of mortality comparing no bolus and bolus.

**Figure 3:**
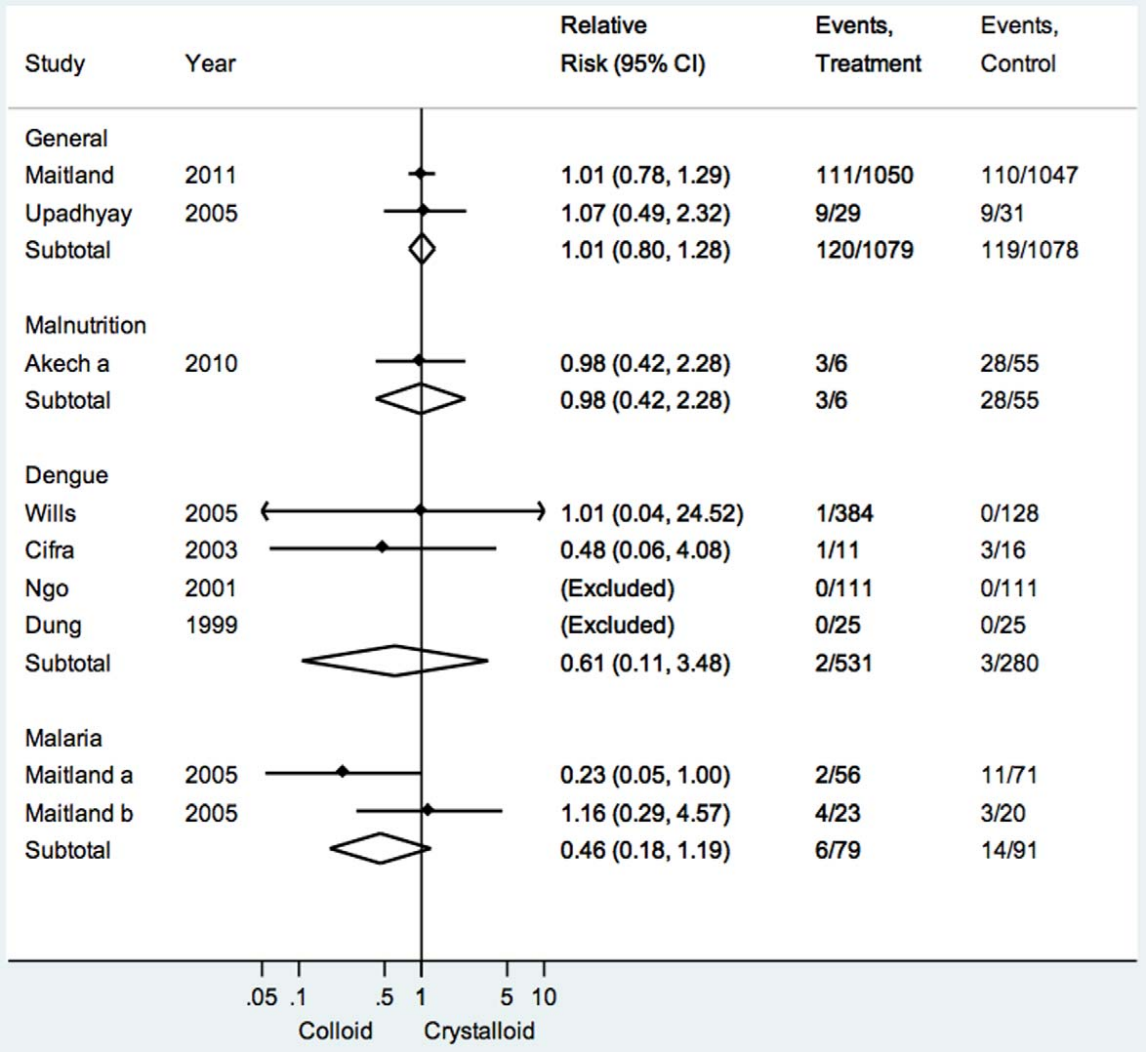
Forest plot for the outcome of mortality comparing colloids and crystalloids.

Four trials assessed interventions in children in septic shock [8], [17]–[19]. Overall mortality across all studies was 10.7%. These studies provide evidence on four comparisons: no bolus vs colloid bolus (2094 patients), no bolus vs crystalloid bolus (2091 patients), colloids bolus vs crystalloid bolus (2157 patients), and different formulations of crystalloid bolus (160 patients). The only significant effect was found in the FEAST trial, the only large trial to compare no bolus vs bolus; in this trial the no bolus group (control) had a lower mortality compared to the bolus group (RR 0.69; 95% CI 0.54–0.89 [Figure 2]). There was no other difference in mortality comparing crystalloid vs colloid (RR 1.01; 95%CI 0.80–1.28; *I* ^2^ 0%; p = 0.9 [Figure 3]).

Four trials (378 patients) assessed interventions in children with shock associated with malaria infection [20]–[23]: a further 1793 children in the FEAST trial had malaria. Overall mortality across all studies was 16.4%. The studies provide evidence comparing bolus and no bolus (2005 patients), colloids vs crystalloids (118 patients), crystalloids vs maintenance therapy (133 patients), and different formulations of colloid (167 patients). For this subgroup of patients, no bolus was found to decrease mortality compared to bolus (RR 0.64; 95%CI 0.45–0.91 [Figure 2]). Heterogeneity was low (*I* ^2^ 0%; p = 0.7). This finding was unchanged using the random-effects method (RR 0.65; 95%CI 0.46–0.92). No statistically significant difference was found for any other comparison (Figure 3).

Four trials (811 patients) assessed colloids vs crystalloids for the treatment of children in dengue shock [24], [26]–[28]. Overall mortality was low at 1.3%. There was no difference in treatment effects across arms (pooled RR 0.61; 95%CI 0.11–3.48). Heterogeneity was low (*I* ^2^ 0%; p = 0.7 [Figure 3]).

One trial was identified assessing the role of fluids in children with severe acute malnutrition (61 patients, of whom 21 had sepsis) [25]. This trial had high mortality (50.8%), but found no difference between study groups which compared an isotonic crystalloid (Ringer’s lactate) against two hypotonic crystalloids (human albumin solution [HAS] or HSD/5D; RR 0.98; 95%CI 0.42–2.28 [Figure 3]).

Although the FEAST trial excluded children with severe malnutrition, 70 (2%) children had a mid-upper-arm circumference (MUAC) ≤11.5 cm (indicating severe acute malnutrition). The effect of bolus fluids was not significantly different in children with a mid-upper arm circumference of >11.5 cm (p = 0.96) [29].

### Secondary Outcomes

Only one study, the FEAST trial, reported mortality at four weeks. Overall, no bolus was protective against mortality compared to bolus (RR 0.69; 95%CI 0.54–0.87). Adverse clinical events, reported by all studies, were low, irrespective of intervention, and ranged from 0% to 11.1% (95%CI 4.2–22.6).

### Interpretation

The majority of all randomized evidence to date comes from the FEAST trial, which found that fluid boluses were harmful compared to no bolus. The 2008 Surviving Sepsis Campaign Guidelines, informed by a modified Delphi process, graded the current paediatric recommendation (20 mL/kg boluses over 5–10 minutes up to 60 mL/kg) as 2C, indicating a weak recommendation with low quality of evidence [30]. Although a single trial, the evidence provided by FEAST that fluid bolus are harmful compared to no bolus is of high quality and sufficient precision, suggesting that the withholding of bolus fluids should be considered for populations similar to those enrolled in FEAST. The important question is the extent to which these results are applicable to other populations. The FEAST trial excluded patients likely to have fluid loss either through bleeding/burns or dehydration due to gastroenteritis. Malnourished children were also excluded. The study population did not include neonates nor children with dengue fever. Caution must be taken in extrapolating the findings beyond populations similar to those included in this trial.

Much of the debate around the validity of the results of this trial have focused on the applied definition of septic shock [9], [14]. Part of the difficulty rests on the fact that there are no uniform definitions for septic shock and many guidelines lack stringent criteria. A re-analysis of the trial data found the results to be robust to the application of different definitions of shock, and while only 65(2%) of children fulfilled the strict WHO definition of shock, even in this small subset there was a significant excess risk associated with boluses with an absolute risk difference of 28% (95%CI 3.4–52.5) [29].

Across all populations, there is no evidence that colloids are superior to crystalloids. A previous systematic review of this question published in 2010 reported outcomes from nine studies (1230 children) and concluded that the evidence base is limited [7]. This systematic review adds to the findings of the previous review principally by including data from the FEAST trial, which increases confidence in this conclusion within the limits of external validity. Of note, more than half of patients enrolled in FEAST had malaria, and overall mortality rates were similar in FEAST to other malaria trials. For other specific populations such as Dengue shock and shock associated with malnutrition, the evidence-base remains limited. Nevertheless, the high rates of survival demonstrated by the Dengue trials provides moderate evidence to support fluid resuscitation for these patients.

There are several strengths and limitations to note. Strengths include the restriction of inclusion of comparative trials and the large meta-analytic dataset compared to previous reviews. Limitations of the evidence-base include the small sample sizes resulting in poor precision for specific populations, in particular malnourished children. In addition, there is inconsistent reporting of secondary outcomes, which limited analysis, although the most important outcomes (mortality and adverse clinical events) were reported by all studies. Few trials included a control group making it impossible to assess the impact of the bolus itself in most trials. Subgroup analyses carry the risk of spurious findings, but these analyses were limited in number and pre-specified in the research protocol. Another potential limitation of this review is the search strategy (only 3 databases searched). Attempts were made to limit the possibility of having missed studies by using a highly sensitive search strategy and consulting with experts in the field. Publication bias is an ever present risk of any systematic review. We were unable to assess publication bias formally due to the limited number of studies identified for review, but the results do not appear to suggest publication bias. Finally, as highlighted by the debate generated following the publication of the FEAST trial, the lack of a standardised definition for shock is an important caveat to consider when comparing different studies. Nevertheless, the results of the FEAST trial were found to be robust to a range of sensitivity analyses that applied different definitions of shock [29].

The most important direction for future research is the applicability of the findings of the FEAST trial to other populations and settings. Simple algorithms are needed to support health-care providers in the triage of patients to determine who could be potentially be harmed by the provision of bolus fluids, and who will benefit. Further studies are urgently needed in the area of shock associated with malnutrition, because the evidence base is scant and mortality high. Work is needed to establish a uniform definition of shock to assist in the comparability of future studies and the application of practice guidelines. The FEAST trial used relatively modest fluid boluses in both rate and time of infusion compared to the other trials included and in comparison to current guidelines. Despite these conservative volumes and rates, bolus therapy was still found to be harmful. Finally, more research is needed to determine exactly what role for fluid therapy in this population beyond the use of bolus therapy.

### Conclusions

The majority of all randomized evidence to date comes from a single trial, which found that fluid boluses were harmful compared to no bolus. While this finding cannot be applied broadly, it does provide for the first time strong evidence on which to base guidelines for management of paediatric septic shock. In the subpopulation of children with haemorrhagic dengue fever, there is moderate evidence to support fluid resuscitation. A priority for future operational research, therefore, is the definition of practical guidelines and algorithms that will allow health-care providers to distinguish between those groups of children likely to benefit from fluid bolus, and those children who could be harmed by this intervention.

## Supporting Information

File S1 **Protocol.**
(PDF)

File S2 **Assessment of Methodological Quality Table.**
(DOC)

File S3 **GRADE evidence profile.**
(DOC)
