# Supplementary material for: Mortality after Fluid Bolus in Children with Shock Due to Sepsis or Severe Infection: A Systematic Review and Meta-Analysis
Source: PLoS One. 2012 Aug 30;7(8):e43953. doi: 10.1371/journal.pone.0043953 (PMC3431361; doi:10.1371/journal.pone.0043953)
Supplement: File S1 — Protocol. (PDF) [file pone.0043953.s001.pdf]

Systematic review

Fluid therapy for the treatment of children with sepsis or severe infection

OBJECTIVE

The objective of this study is to assess the relative benefit of fluid therapy for children with sepsis or severe infection. In the primary review, comparative studies of fluid therapy vs control will be sought, and subject to GRADE evidence assessment. Comparative studies of different fluid regimens will be also be assessed.

METHODS

Search Strategy

- See annex

Databases

- MEDLINE via PubMed
- EMBASE
- Cochrane Central Register of Controlled Trials (CENTRAL)

Other

- Bibliographies of all relevant articles will be checked for further studies
- The final list of inclusions will be crosschecked with experts to determine the existence of potentially eligible studies

Inclusions and exclusions

Types of studies

- Randomized or Quasi-Randomized Trials
- Controlled Before and After Studies
- All other study types (non-comparative observational studies, non-systematic observations) will be excluded

Types of participants

Inclusions:

- Children with shock, defined according to study criteria

Exclusions:

- Non-human studies
- Adults
- Non-septic causes of shock (eg burns)

- Fluid refractive shock

Types of interventions

- Fluid therapy of any kind
- Where appropriate, outcomes will be stratified by type of therapy

Types of outcomes

Primary

- Mortality at 48 hours

Secondary

- Mortality at 4 weeks
- Adverse clinical events

DATA ANALYSIS

Summary estimates

Primary and secondary outcomes will be represented as proportions, and as relative risks, using intent-to-treat data. If appropriate, relative risks will be pooled using random-effects meta-analysis.

Assessment of study quality and risk of bias

- The Grade approach will be used to assess study quality and risk of bias

Assessment of heterogeneity

- It is possible for some outcomes that meta-analysis may be conducted. If it is, we will examine heterogeneity by using the χ^2 statistic with a significance level of >0.10, and the I^2 statistic. We will interpret an I^2 estimate greater than 50% as indicating moderate or high levels of heterogeneity and will investigate its causes.

Assessment of reporting bias

- Where sufficient studies are available, we will assess publication visually using funnel plots.

Subgroup analyses

- Subgroup analyses will explore differences according to baseline co-morbidities as these may influence patient outcomes

Annex: search strategy for Pubmed

#43	**Search** #26 AND #42
#42	**Search** #34 OR #41
#41	**Search** #35 OR #36 OR #37 OR #38 OR #39 OR #40
#40	**Search** Prospective study[MeSH Terms]
#39	**Search** Non-randomized study
#38	**Search** Comparative study
#37	**Search** comparative before after
#36	**Search** cohort
#35	**Search** comparative cohort
#34	**Search** #27 OR #28 OR #29 OR #30 OR #31 OR #32 OR #33
#33	**Search** meta analysis
#32	**Search** cluster random*
#31	**Search** clinical trial
#30	**Search** single blind procedure
#29	**Search** double blind procedure
#28	**Search** random*[Title/Abstract]
#27	**Search** randomization[Title/Abstract]
#26	**Search** #12 AND #19 AND #25
#25	**Search** #20 OR #21 OR #22 OR #23 OR #24
#24	**Search** IV fluid*[Title/Abstract]
#23	**Search** intravenous fluid*[Title/Abstract]
#22	**Search** fluid management[Title/Abstract]
#21	**Search** fluid resuscitation[Title/Abstract]
#20	**Search** fluid therapy[MeSH Terms]
#19	**Search** #13 OR #14 OR #15 OR #16 OR #17 OR #18
#18	**Search** Adolescent*[Title/Abstract]
#17	**Search** pediatr*[Title/Abstract]
#16	**Search** paediatr*[Title/Abstract]
#15	**Search** paediatric*[Title/Abstract]
#14	**Search** infant*[Title/Abstract]
#13	**Search** child*[Title/Abstract]
#12	**Search** #1 OR #2 OR #3 OR #4 OR #5 OR #6 OR #7 OR #8 OR #9 OR #10 OR #11
#11	**Search** pneumonia[MeSH Terms]
#10	**Search** dengue shock syndrome[Title/Abstract]
#9	**Search** dengue shock syndrome[MeSH Terms]
#8	**Search** meningitis[Title/Abstract]
#7	**Search** meningitis[MeSH Terms]
#6	**Search** malaria[Title/Abstract]
#5	**Search** malaria[MeSH Terms]
#4	**Search** sepsis[MeSH Terms]
#3	**Search** febrile illness[Title/Abstract]
#2	**Search** shock[Title/Abstract]
#1	**Search** septic shock[MeSH Terms]
