# Supplementary material for: Mortality after Fluid Bolus in Children with Shock Due to Sepsis or Severe Infection: A Systematic Review and Meta-Analysis
Source: PLoS One. 2012 Aug 30;7(8):e43953. doi: 10.1371/journal.pone.0043953 (PMC3431361; doi:10.1371/journal.pone.0043953)
Supplement: File S2 — Assessment of Methodological Quality Table. (DOC) [file pone.0043953.s002.doc]

**Supporting Information File S2: Assessment of Methodological Quality Table**

| Study | Allocation concealment | Loss to follow up <20% | Adverse event reported |
| --- | --- | --- | --- |
| Maitland et al, 2011 | Yes | Yes | Yes |
| Chopra et al, 2011 | No | Yes | Yes |
| Santhanam et al, 2008 | Yes | Yes | Yes |
| Upadhyay et al, 2005 | Yes | Yes | No |
| Akech et al, 2010b | Yes | Yes | Yes |
| Akech 2006 | No | Yes | Yes |
| Maitland et al 2005a | Yes | Yes | Yes |
| Maitland et al, 2005b | No | Yes | Yes |
| Wills et al, 2005 | Yes | Yes | Yes |
| Cifra et al, 2003 | No | Yes | No |
| Ngo et al, 2001 | Yes | Yes | Yes |
| Dung et al, 1999 | Yes | Yes | Yes |
| Akech et al, 2010a | Yes | Yes | Yes |
