# Supplementary material for: Mortality after Fluid Bolus in Children with Shock Due to Sepsis or Severe Infection: A Systematic Review and Meta-Analysis
Source: PLoS One. 2012 Aug 30;7(8):e43953. doi: 10.1371/journal.pone.0043953 (PMC3431361; doi:10.1371/journal.pone.0043953)
Supplement: File S3 — GRADE evidence profile. (DOC) [file pone.0043953.s003.doc]

**Supporting Information File S3: GRADE evidence profile**

| Population | Design | Limitations | Inconsistency | Indirectness | Imprecision | Quality |
| --- | --- | --- | --- | --- | --- | --- |
| Septic shock | 4 RCTs | No serious limitations | No serious inconsistency | No serious indirectness | No serious Imprecision | High |
| Malaria | 4-5 RCTs* | No serious limitations | No serious inconsistency | No serious indirectness | No serious Imprecision | Moderate/High |
| Dengue | 4 RCTs | No serious limitations | No serious inconsistency | No serious indirectness | Serious Imprecision** | Moderate |
| Malnutrition | 1 RCT | No serious limitations | Not applicable | No serious indirectness | Serious Imprecision*** | Low |

*Assessment based on the potential application of the FEAST trial to children with malaria

**Due to low event rate in trials

***single trial of small sample size
